# Chronically altered NMDAR signaling in epilepsy mediates comorbid depression

**DOI:** 10.1186/s40478-021-01153-2

**Published:** 2021-03-24

**Authors:** Mohammad Amin Sadeghi, Sara Hemmati, Sina Mohammadi, Hasan Yousefi-Manesh, Ali Vafaei, Meysam Zare, Ahmad Reza Dehpour

**Affiliations:** 1 Brain and Spinal Cord Injury Research Center, Neuroscience Institute, Tehran University of Medical Sciences, Tehran, Iran; 2 Experimental Medicine Research Center, Tehran University of Medical Sciences, Tehran, Iran; 3 School of Medicine, Tehran University of Medical Sciences, Tehran, Iran; 4 Students’ Scientific Research Center, Tehran University of Medical Sciences, Tehran, Iran; 5 School of Pharmacy, Tehran University of Medical Sciences, Tehran, Iran; 6 Department of Physiology, Faculty of Medical Sciences, Tarbiat Modares University, Tehran, Iran; 7 Department of Pharmacology, School of Medicine, Tehran University of Medical Sciences, Tehran, Iran

**Keywords:** Depression, Epilepsy, Intracellular signaling, Nitric oxide, Nitrosative stress, N-methyl-D-aspartate receptors, MAP kinase signaling system, Nitric oxide synthase type I, Brain-derived neurotrophic factor, Immediate early genes

## Abstract

Depression is the most common psychiatric comorbidity of epilepsy. However, the molecular pathways underlying this association remain unclear. The NMDA receptor (NMDAR) may play a role in this association, as its downstream signaling has been shown to undergo long-term changes following excitotoxic neuronal damage. To study this pathway, we used an animal model of fluoxetine-resistant epilepsy-associated depression (EAD). We determined the molecular changes associated with the development of depressive symptoms and examined their response to various combinations of fluoxetine and a selective neuronal nitric oxide synthase inhibitor, 7-nitroindazole (NI). Depressive symptoms were determined using the forced swim test. Furthermore, expression and phosphorylation levels of markers in the ERK/CREB/ELK1/BDNF/cFOS pathway were measured to determine the molecular changes associated with these symptoms. Finally, oxidative stress markers were measured to more clearly determine the individual contributions of each treatment. While chronic fluoxetine (Flxc) and NI were ineffective alone, their combination had a statistically significant synergistic effect in reducing depressive symptoms. The development of depressive symptoms in epileptic rats was associated with the downregulation of ERK2 expression and ELK1 and CREB phosphorylation. These changes were exactly reversed upon Flxc + NI treatment, which led to increased BDNF and cFOS expression as well. Interestingly, ERK1 did not seem to play a role in these experiments. NI seemed to have augmented Flxc’s antidepressant activity by reducing oxidative stress. Our findings suggest NMDAR signaling alterations are a major contributor to EAD development and a potential target for treating conditions associated with underlying excitotoxic neuronal damage.

## Introduction

Epilepsy is associated with a variety of neurological comorbidities, the most common of which is depression [17]. Unfortunately, depression in epilepsy remains underdiagnosed and undertreated, possibly due to fears of proconvulsant effects of antidepressants and pharmacokinetic interactions between antidepressants and anti-epileptic drugs (AEDs) [23, 48, 55]. Interestingly, a bidirectional relationship exists between epilepsy and depression, suggesting common pathophysiological pathways between the two conditions [24]. Modulation of these pathways may provide more effective ways of treating epilepsy-associated depression (EAD) than targeting each condition separately. In this study, we have used an animal model of EAD to uncover these common pathways [31].

In this model, LiCl-pilocarpine induced status epilepticus (SE) is used to prime epileptogenesis in rats. It has been shown that depressive-like symptoms develop following epileptogenesis and that these symptoms are resistant to fluoxetine treatment [31]. Importantly, epileptogenesis clearly precedes development of depression in this model. Therefore, identification of the pathways responsible for this pattern may shed light on the common pathophysiological elements between epilepsy and depression. As described in detail in the Methods section, we modified the LiCl-pilocarpine SE model by Mazarati et al. [31] to reduce mortality rates due to SE. Nevertheless, the characteristic development of fluoxetine-resistant EAD in the original model remained unchanged.

In previous studies, various treatments such as an interleukin-1 receptor antagonist [40]; the N-methyl-D-aspartate receptor (NMDAR) NR2B subunit antagonist, ifenprodil [39]; and a soluble epoxide hydrolase inhibitor, 1-trifluoromethoxyphenyl-3-(1-propionylpiperidin-4-yl) urea [46], have been shown to effectively alleviate depression-like behavioral deficits in EAD. Here we aimed to more specifically and comprehensively characterize the pathway underlying the fluoxetine-resistant depressive symptoms observed in this model by a) targeting a downstream secondary messenger in the NMDAR signaling pathway; and b) implementing a study design to find clear evidence of synergism and positive interaction between our proposed treatment and fluoxetine in behavioral and molecular studies.

To overcome resistance to fluoxetine, we chose to combine it with the selective neuronal nitric oxide synthase (nNOS) inhibitor, 7-nitroindazole (NI). Neuronal NOS is localized to post synaptic NMDAR by the scaffold protein, PSD-95 [8]. The influx of calcium upon NMDAR activation stimulates nitric oxide (NO) production by nNOS in a phosphorylation- and calmodulin-dependent manner [44, 60]. Therefore, NO constitutes a secondary messenger system for NMDAR. Importantly, while the LiCl-pilocarpine SE model initially targets M1 receptors through cholinergic activation, SE continuation, neuronal lesion formation, and development of spontaneous seizures have been shown to result from glutamatergic activation [33]. This excessive glutamate signaling has been shown to induce neuronal death and, therefore, is dubbed glutamate excitotoxicity. Nitric oxide is an important mediator of these excitotoxic effects [26]. In physiological conditions, nNOS is inhibited shortly after activation by calcium/calmodulin-dependent kinase II (CaMKII), which itself is activated by NMDAR. This ensures that NO is produced only transiently after NMDAR activation. However, following excitotoxic activity such as prolonged seizures (i.e., SE), nNOS becomes constitutively activated [44]. Indeed, this may be due to the inhibition of CaMKII in excitotoxic states which remains for up to 6 weeks after LiCl-pilocarpine SE induction [12]. In agreement with these findings, nitric oxide levels have been shown to increase 30 min after LiCl-pilocarpine SE and remain at high levels for up to 7 days [29]. As a result, we hypothesized that the chronically increased NO may play a role in not only the initial stages of neuronal damage, but also epileptogenesis and the development of depressive-like symptoms afterwards.

After initial confirmation of synergistic antidepressant effects for the NI and fluoxetine combination in behavioral studies, we set out to identify the underlying molecular mechanisms for this interaction. The Ras/extracellular-regulated kinase (ERK)/cAMP-response element binding protein (CREB)/brain-derived neurotrophic factor (BDNF) pathway is one of the most well studied pathways in the nervous system. In physiological conditions, this pathway is directly activated by NMDAR in both NO/guanylyl cyclase (GC)/cGMP-dependent protein kinase (PKG)-dependent and independent manners in the context of cellular processes such as long-term potentiation (LTP) and neurogenesis [52]. This also holds true immediately after pathological states such as seizures, brain ischemia, and traumatic brain injury which are followed by a rapid phase of neurogenesis and mossy fiber sprouting [28, 30, 36]. In the chronic epileptic state, however, this pattern of signaling is altered. Previous studies have shown that the regulatory subunit composition of NMDAR shifts from NR2A to NR2B in chronic epilepsy [21, 39]. Unlike NR2A, NR2B has been shown to exert a biphasic control of the Ras/ERK/CREB/BDNF pathway by stimulating and inhibiting ERK1/2 and CREB phosphorylation under physiological and pathological conditions, respectively [52]. These findings brought us to expect a downregulation of this pathway in the chronic epileptic state in our model. In addition to epilepsy, this pathway has been studied extensively in depression, where it is found to be downregulated in the prefrontal cortex and hippocampus of depressed rats. Furthermore, many antidepressant compounds have been shown to exert their therapeutic effects through reversing this downregulation [59]. Based on these findings, we hypothesized epilepsy-induced downregulation of this pathway to play a role in depression pathogenesis. In addition to CREB, we studied ELK1 as another transcription factor downstream to ERK. Importantly, while ELK1 is generally activated alongside CREB upon ERK activation [13, 57], it has recently been shown to independently mediate depressive behaviors [3]. Therefore, we studied the activation of both CREB and ELK1 to account for any instances of differential regulation of the two transcription factors. cFOS, the pre-eminent immediate early gene (IEG), was used as a marker of ELK1 activation [7].

Following confirmation that the synergistic effects of fluoxetine and NI may converge into ERK/CREB/ELK1/BDNF/cFOS activation, we set out to more clearly identify the mechanisms underlying this convergence. While fluoxetine has been shown to increase ERK/CREB activity through multiple pathways, namely through 5-HT1a and CaMKIV activation [2, 50, 54], NO has been shown to exert a biphasic effect depending on its concentration. At low concentrations, NO is neuroprotective and functions in physiological signaling, mostly in a GC/cGMP/PKG-dependent manner. At high concentrations, however, NO has been shown to participate in mitochondrial function impairment, induction of oxidative and nitrosative stress, and S-nitrosylation (directly) and tyrosine nitration (through conversion to peroxynitrite) of proteins [9, 51]. Since mitochondrial impairment and oxidative stress are well known elements in the pathophysiology of epilepsy and depression [4, 33, 58], we hypothesized that modulation of this oxidative stress and restitution of mitochondrial function was how NI enabled activation of the ERK/CREB/BDNF signaling pathway. Therefore, as the final step, we studied changes in indicators of oxidative stress in the study groups as well.

## Methods and materials

### Animals

Male Wistar rats weighing more than 250 g were used in all experiments. All animals were maintained under standard housing conditions of temperature (21–23 °C), humidity (55%), and light/dark cycle (12-h light/dark), and had access to food and water ad libitum. All experiments were performed in accordance with the standards of animal care determined by the Council of Laboratory Animals of the Experimental Medicine Research Center, Tehran University of Medical Sciences, Tehran, Iran (Approval No. IR.TUMS.VCR.REC.1396.2457).

### Model

We have used a modified version of the model presented by Mazarati et al. [31] with the modifications intended to reduce mortality rates [35]. On the day before SE induction, the animals received an intraperitoneal (i.p.) injection of 130 mg/kg LiCl (Sigma, St. Louis, MO, USA). 23.5 h later, they were injected subcutaneously (s.c.) with 10 mg/kg hyoscine butylbromide to reduce the peripheral cholinergic effects of pilocarpine and received 40 mg/kg pilocarpine s.c. (Sigma) 30 min later (day 0). The animals were observed until the development of convulsive activities which were ranked based on Racine’s scale [42]. Animals showing continuous seizure activity that reached severity ratings of > 3 were characterized as undergoing status epilepticus. If seizure severity remained < 4 within 45 min of injection, another dose of 40 mg/kg pilocarpine s.c. was injected. Similarly, a third dose was used if required; however, animals failing to show signs of SE following the third dose were excluded from the study (n = 14). Immediately after initiation of SE, the animals received an intramuscular (i.m.) injection of 2.5 mg/kg xylazine (Sigma) to reduce injury potential due to clonic contractions. The rats were maintained in xylazine-modified SE for 3 h and sterile vitamin A eye ointment was used to prevent drying of the eyes. SE was stopped after 3 h with simultaneous i.p. injections of 10 mg/kg diazepam and 50 mg/kg phenytoin. If the animal continued to show seizure activity after 30 min, another dose of diazepam was injected. SE was successfully stopped in all animals following these injections. Following the cessation of SE and up to 3 days after induction, the animals were cared for with fluid resuscitation (pre-warmed 0.9% saline s.c. and i.p.) to accelerate recovery and reduce mortality. The Sham group (n = 9) received all injections except for pilocarpine for which they received 0.9% saline (s.c.) instead. In total, 84 rats underwent SE induction. 14 of these animals failed to reach stage 4–5 seizures and were excluded from the study. Of the remaining 70, 48 (68.6 percent) survived for further analysis.

### Study design

Of the 48 rats which had survived the SE phase, 6 and 3 were used for recording of spontaneous seizures and histological studies, respectively, and the rest were used for the main study. Similarly, 3 of the Sham group were used for histological studies while the rest (6 rats) were used for the main study. The experimental structure of the main study was as follows. 45 days after SE induction, initiation of epilepsy-associated depression was confirmed using the forced swim test (FST1). FST1 was preceded by a 15-min pretest the day before. The value of mean plus two standard deviations of immobility times in FST1 for the Sham group was used as the EAD cutoff (17.33 + 2 × 9.29). From the 48 rats entering the main study, 6 had not developed EAD by day 45 and were excluded from the study. The animals that were determined to be depressed following FST1 received 6 different drug combinations in two phases: chronic and acute. In the chronic phase, they underwent a 10-day period of daily i.p. injections of either 10 mg/kg fluoxetine (Sigma) (chronic fluoxetine–Flxc) or 0.9% saline starting from the day after FST1. Such daily injections have been shown to produce stable concentrations of fluoxetine and its active metabolite, norfluoxetine, in brain tissue [19]. On the 10th day, in addition to the final injection of their chronic treatment, the rats received an acute treatment 30 min prior to FST2. The acute treatment consisted of two i.p. injections of saline, and/or 15 mg/kg 7-nitroindazole, and/or 10 mg/kg fluoxetine (acute fluoxetine–Flxa). For all injections, 7-nitroindazole was suspended in saline with 5% DMSO and fluoxetine was solved in saline and heated to 60 °C for complete solvation. In total, 7 groups were present in this study. The treatment combinations for each group are presented in Table 1.

**Table 1:** Study groups and treatment combinations

Group	SE induction	Chronic phase (daily i.p. injections for 10 days)	Acute phase (two i.p. injections 30 min prior to FST2)
Sham	No	saline	saline + saline
Saline	Yes	saline	saline + saline
NI	Yes	saline	15 mg/kg NI + saline
Flxa	Yes	saline	saline + 10 mg/kg Flx
Flxa + NI	Yes	saline	15 mg/kg NI + 10 mg/kg Flx
Flxc	Yes	10 mg/kg Flx	saline + saline
Flxc + NI	Yes	10 mg/kg Flx	15 mg/kg NI + saline

Unlike FST1, FST2 was not preceded by a 15-min pretest the day before. However, both FST1 and FST2 were conducted after a seizure-free period of at least 4 h was observed for all animals to prevent any immediate effects of seizures on test results. Immediately after FST2, the animals were anesthetized using dietyhyl ether (Supelco, Bellefonte, PA, USA) and decapitated. The animals’ heads were placed on an ice bed and the brains were quickly extracted and dropped into ice-cold normal saline for 45 s. Then, the hippocampi were rapidly extracted on an ice-cold surface and were flash-frozen in liquid nitrogen. After the flash-freeze, the hippocampi were transferred to a − 80 °C freezer awaiting further analysis.

### Recording of spontaneous seizures

To confirm the development of epilepsy in our modified model, a group of 6 post-SE rats were studied for spontaneous seizures using video monitoring and local field potential (LFP) recordings from the dorsal hippocampus. 20 days after SE, the rats underwent surgery for electrode placement. Under anesthesia with 100 mg/Kg ketamine (10%, Alfasan, The Netherlands) and 10 mg/Kg xylazine (20%, Alfasan, The Netherlands), the animals underwent stereotaxic implantation of a monopolar recording electrode in the CA1 region of right dorsal hippocampus (coordinates: A, − 3.2 mm; L, 2 mm; and V, 2.3 below dura) [38]. The recording (stainless steel, Teflon coated, 127 µm in diameter, A.M. Systems, USA) and reference (connected to the skull by a miniature screw) electrodes were insulated except at their tips. Three screws were planted into the skull as anchors. All electrodes were connected to pins of a lightweight multichannel miniature socket as a head-stage and were fixed on the skull with dental acrylic. The animals were allowed to recover for 10 days after the surgery before initiation of experiments.

**Fig. 1:**
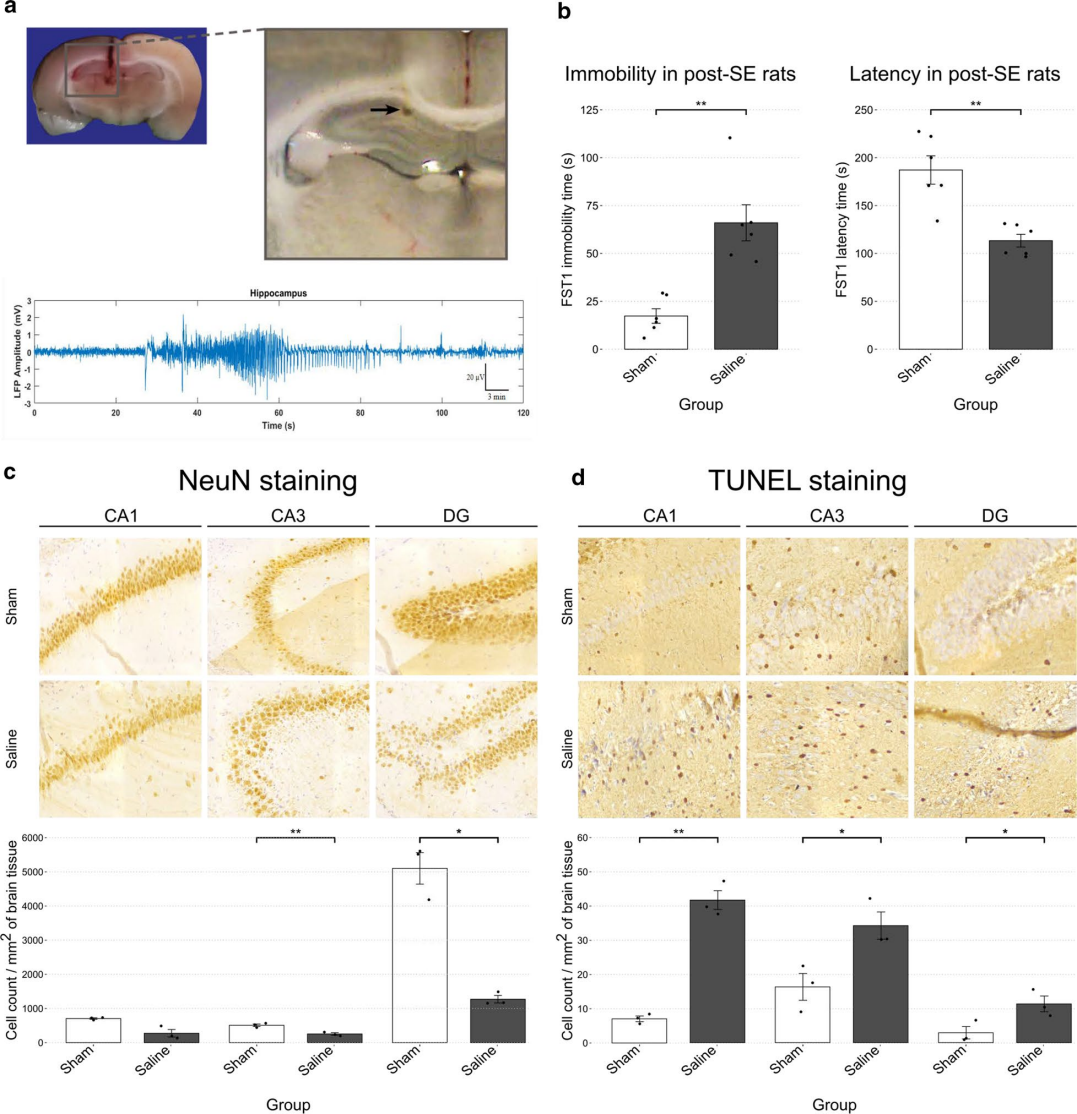
Validation of EAD development in post-SE rats. **a** The hippocampal insertion site of the LFP electrode and a sample recording of one of the spontaneous seizures observed 30 days after SE induction. **b** Comparison of depressive symptoms in the FST between post-SE (Saline) and Sham rats. **c** & **d** Comparison of the number of NeuN-immunoreactive (**c**) and TUNEL-stained (**d**) cells in 3 subregions of the hippocampus: CA1, CA3, and DG; between post-SE (Saline) and Sham rats. All comparisons were done using the Welch’s t-test and statistical significance is shown as follows: *p < .05, **p < .01

After recovery, hippocampal LFPs were recorded when the animals were put in a plexiglass recording box inside a Faraday’s cage. The rat’s head-stage was connected to a flexible, shielded cable and the animal was allowed to move freely during the recording. Signals were filtered at 3 kHz, amplified and digitized (at 10 kHz) using a PC-based data acquisition system (BIODAC ES1721, TRITA WaveGram CO., Tehran, Iran) and were continuously monitored and stored on disk. Hippocampal LFPs were recorded continuously for one week. The behaviors of the animals were also video recorded during this one-week period for verification of seizure events. Electrode placement and a representative seizure recording are demonstrated in Fig. 1a. 5 out of the 6 rats showed at least one spontaneous seizure throughout this period (minimum = 1, maximum = 3, median = 1), confirming the development of a chronic epileptic state following SE induction in our model at rates consistent with previous studies [31, 39]. Importantly, while we did not perform monitoring for all animals in this study, all rats were initially screened for depressive symptoms using the forced swim test before initiation of experiments as described in the Study Design section. All animals that had developed depressive symptoms were assumed to have underlying epilepsy. As a result, although our observations during the study supported this assumption, we cannot rule out with certainty the possibility of development of depressive symptoms without development of spontaneous recurrent seizures after SE.

### Histology

On day 45, 3 rats from each of the post-SE (Saline) and Sham groups underwent FST to ensure development of EAD. Following confirmation, the rats were anesthetized with diethyl ether and were then perfused with ice-cold PBS followed by 4% paraformaldehyde. The brains were dissected and stored in 4% paraformaldehyde solution for 18 h at 4 °C and cryoprotected in 30% sucrose solution. Coronal brain cuts (20 um) were performed 4.3 mm posterior to the Bregma for the dorsal hippocampus.

For NeuN immunohistochemistry, sections were treated with PBS solution containing 20% methanol and 3% hydrogen peroxide for 20 min. The sections were further rinsed in PBS, incubated for 15 min in 1% sodium borohydride, and blocked with 10% normal horse serum in PBS containing 0.1% Triton X-100. Then, the sections were incubated overnight with an anti-NeuN monoclonal mouse antibody (1:1000; Chemicon, Billerica, MA, USA). After rinsing, the sections were incubated with secondary biotin-coupled anti-mouse antibodies (1:200; Vector Laboratories, Burlingame, CA, USA) for 1 h. To ensure the specificity of NeuN immunoreactivity, control staining was performed without the primary antibodies. Photomicrographs of CA1, CA3, and dentate gyrus (DG) subfields of the hippocampus (10x) were captured with the CELLNAMA Slide Scanner. NeuN-positive neurons were counted in two sections of each region (350 × 350 um^2^) with 120‑µm intervals using the ImageJ software and were averaged for each animal.

For TUNEL assay, paraffin sections were de-waxed, rehydrated in a graded series of alcohol, and rinsed with distilled water. Then, the Slides were incubated in proteinase K working solution (20 μg/mL in 10 mM Tris/HCl, pH 7.4–8.0; Roche Diagnostics, Mannheim, Germany) for 15 min at room temperature. Further steps were performed according to the instructions provided by the manufacturer. Cell counting was performed on two randomly selected non-overlapping subfields in the CA1, CA3, and DG regions of the hippocampus, survival/apoptosis index was calculated for each subfield, and the results were averaged for each animal. Condensed nuclei were taken to indicate TUNEL-positive apoptotic neurons.

### Forced swim test

For the forced swim test (FST), an acrylic cylinder (60 cm in height and 30 cm in width) was filled up to 45 cm with water maintained at 23–25 °C. The cylinder was washed and its water was replaced between each test. 24 h prior to the first test day (FST1), the rats were habituated to the FST conditions with a 15-min preswim [49]. For the test, animals were placed in the cylinder for 5 min and were filmed. An observer blind to the experimental conditions later analyzed the videos and calculated total immobility time and latency time to first immobility for each animal.

### Measurement of oxidative stress markers

#### Nitric oxide

Nitrite levels were measured in the rat hippocampi as a proxy of NO using the Griess method (Navand Salamat, Urmia, Iran). Briefly, equal masses of hippocampi were homogenized in PBS and were then centrifuged to remove tissue debris. Using the protocol described by the manufacturer, all proteins were preciptitated from the supernatants and aliquots of the remaining protein-less supernatant were reacted with the same volume of Griess reagent. After a 10-min incubation period, nitrite concentration was quantified spectrophotometrically at 570 nm with regard to a standard curve plotted based on known-concentration standards provided by the manufacturer. Samples from three animals in each study group were used for these experiments.

#### Reduced glutathione

Reduced glutathione (GSH) was measured using DNTB (Navand Salamat). Tissue homogenates were centrifuged at 10000 g for 10 min at 4 °C and the supernatants were collected. DTNB and NADPH were added to the samples, resulting in a yellowish color due to DNTB’s reaction with thiol compounds. Optical densities were measured at 412 nm and the concentrations were calculated based on the standard samples provided by the manufacturer. Samples from three animals in each study group were used for these experiments.

#### Malondialdehyde

Malondialdehyde (MDA) levels were measured as a representative of lipid peroxidation using the thiobarbituric acid (TBA) method (Navand Salamat). Based on the manufacturer’s protocol, tissue homogenates were mixed with TBA reagent to yield colored compounds. Following centrifugation, absorbance of supernatants were measured at 532 nm and MDA concentrations were determined based on the standard curve. Samples from three animals in each study group were used for these experiments.

### Western Blotting

Rat hippocampi were homogenized in lysis buffer consisting of TRIS–HCl, SDS, DTT, glycerol, and NP40. The homogenates were then centrifuged at 15,000 g for 10 min at 4 °C, and the supernatants were used for SDS-PAGE. Ten micrograms of protein was resolved on 10% SDS-PAGE gel and moved onto polyvinylidene difluoride (PVDF) (Millipore, Burlington, MA, USA) membranes. Membranes were blocked for 120 min with 5% non-fat skimmed milk and incubated with the following primary antibodies overnight: GAPDH (sc-32233), ELK1 (sc-365876), pELK1 (sc-8406), and cFos (sc-166940) from Santa Cruz Biotechnology (Dallas, TX, USA); and nNOS (ab15203), ERK1/2 (ab17942), pERK1/2 (ab214362), CREB (ab31387), pCREB (ab32096), and BDNF (ab203573) from Abcam (Cambridge, UK). Membranes were then washed 3 times with TBST (TBS + tween 80) and incubated for 1 h at room temperature with secondary antibodies for mouse (sc-516102, Santa Cruz) and rabbit (ab6721, Abcam) IgG. Bands were visualized using the BM Chemiluminescence Western Blotting Kit (Sigma) and were detected using a gel documentation system. An open-source image-processing program, ImageJ, was used to quantify the optical densities of each band. The relative expressions of proteins were corrected based on their corresponding GAPDH and their fold change was calculated compared to the Saline group. Samples from four animals in each study group were used for these experiments.

When interpreting the pattern of molecular changes observed, we must note that the antibodies used in this study for ERK1/2 and ELK1 targeted the total protein population, while the antibody for CREB targeted only the unphosphorylated subpopulation.

### Statistical Analysis

To validate the development of depressive symptoms in epileptic rats, results from FST1, histological, and western blotting experiments were compared between the Sham and Saline groups using the independent Welch’s t-test. In addition, to assess the effect of repeated FSTs on depressive symptoms, paired t-tests were used on data from FST1 and FST2 for the Sham and Saline groups. Furthermore, the amount of change between FST1 and FST2 was compared between the Sham and Saline groups using the independent Welch’s t-test on change scores defined as (FST2-FST1)/FST1.

The therapeutic effects of the drug combinations used in this study were compared based on their ability to reduce depressive symptoms between FST1 (pre) and FST2 (post). In such pre-post analyses, one could either use ANOVA to compare the amount of change adjusted by the pretreatment values between the groups (such as the change score defined earlier), or use ANCOVA on post-treatment measurements while using the pretreatment values as the covariate. Although both methods have been used in the literature, ANCOVA is generally accepted to be the preferred one [37]. In our study, the underlying assumptions of ANCOVA, as tested using Levene’s test for homogeneity of variance; Breusch-Pagan test for homoscedasticity; Shapiro–Wilk test and Q-Q plots for normality; and regression analysis for homogeneity of regression slopes and error levels based on studentized residuals, were upheld only for immobility times and not for latency times. As a result, for immobility times, we used two-way ANCOVA with immobility times in FST1 as the covariate. Type III analysis of variance was used due to the unbalanced design and our hypothesis regarding the interaction of treatments. Significant interactions were interpreted using simple effect analysis with orthogonal contrasts and effect sizes for pairwise comparisons were calculated using Cohen’s D. Effect sizes in the overall ANCOVA were calculated as partial eta squared [16].

For latency times, the assumptions of homoscedasticity, independence of covariate from experimental combinations, and normality (in chronic latency) were violated. While both robust and nonparametric methods have been proposed for such circumstances, most are only applicable to one-way settings [61]; and the ones applicable in multiway settings have not been extensively validated [14]. As a result, here we opted to analyze change scores of latency times between FST1 and FST2 calculated as (FST2-FST1)/FST1. We conducted a robust trimmed means two-way ANOVA followed by pairwise comparisons of trimmed means as explained by Wilcox [61]. Effect sizes for pairwise comparisons were calculated using Cohen’s D.

The effects of the drug combinations on the results from western blotting and oxidative stress assays were compared using type III two-way ANOVA. Significant interactions were interpreted using simple effect analysis with orthogonal contrasts and effect sizes for pairwise comparisons were calculated using Cohen’s D. Effect sizes in the overall ANOVA were calculated as omega squared [16].

All analyses were conducted using R, version 4.0.2; and all plots were drawn using R and Inkscape, version 1.0.1.

## Results

### Validating the development of EAD and the use of repeated FSTs for its assessment

#### Comparison of depressive symptoms between post-SE and Sham rats

To determine whether post-SE rats developed epilepsy-associated depression, 6 post-SE rats (Saline) were compared to 6 rats from the Sham group using FST1 on day 45. Immobility times were significantly higher in the Saline group (mean = 66, SD = 23.07) compared to the Sham group (17.33, SD = 9.29) using Welch’s t-test (*t*(6.6), *p* = 0.002, *d* = − 2.77). The same was true for latency times as the Saline (mean = 113.33, SD = 16.28) group first became immobile much sooner than the Sham group (mean = 187.17, SD = 36.25) (*t*(6.9), *p* = 0.003, *d* = − 2.63) (Fig. 1b).

#### Comparison of the histological characteristics of post-SE and Sham hippocampi

To assess the neuronal damage caused by our modified model of EAD, we assessed cell count and apoptosis rates in microscopic slides of the rat hippocampi based on NeuN immunoreactivity and TUNEL staining, respectively. The number of NeuN-positive cells were markedly less in the post-SE (Saline) group compared to the Sham group for all three hippocampal regions, with the largest difference being observed in the dentate gyrus (CA1: *t*(2.19) = 3.82, *p* = 0.054, *d* = 3.21; CA3: *t*(4) = 4.83, *p* = 0.008, *d* = 3.95; DG: *t*(2.23) = 8.09, *p* = 0.011, *d* = 6.61) (Fig. 1c). In contrast, TUNEL-positive cells were significantly increased in the Saline group, especially in the CA1 region (CA1: *t*(2.37) = − 12.08, *p* = 0.003, *d* = − 9.86; CA3: *t*(4) = − 3.21, *p* = 0.033, *d* = − 2.62; DG: *t*(3.79) = − 2.87, *p* = 0.048, *d* = 2.34). Therefore, widespread neuronal loss in the hippocampus was accompanied by elevated levels of apoptosis even 45 days after SE-induction (Fig. 1d).

#### Assessment of the effect of repeated FSTs

Following the successful development of depressive symptoms in the post-SE rats, we went on to study whether repeating the FST would affect symptoms. For this, we compared immobility and latency times between FST1 and FST2 for both the Sham and Saline groups using the paired t-test. In the Sham group, immobility times did not differ significantly (*t*(5) = 0.48, *p* = 0.7, *d* = 0.09) between FST1 (mean = 17.33, SD = 9.29) and FST2 (mean = 16.33, SD = 5.28). However, the difference in latency times between FST1 (mean = 187.17, SD = 36.25) and FST2 (mean = 166`0.67, SD = 23.83) was more substantial (*t*(5) = 2.4, *p* = 0.06, *d* = 0.56), showing a decrease in FST2 compared to FST1.

Conversely, in the Saline group, neither immobility (FST1: mean = 66.00, SD = 23.07 / FST2: mean = 61.17, SD = 23.22; *t*(5) = 0.87, *p* = 0.4, *d* = 0.21) nor latency (FST1: mean = 113.33, SD = 16.28 / FST2: mean = 109.17, SD = 18.05; *t*(5) = 0.88, *p* = 0.4, *d* = 0.24) times differed noticeably between FST1 and FST2.

**Fig. 2:**
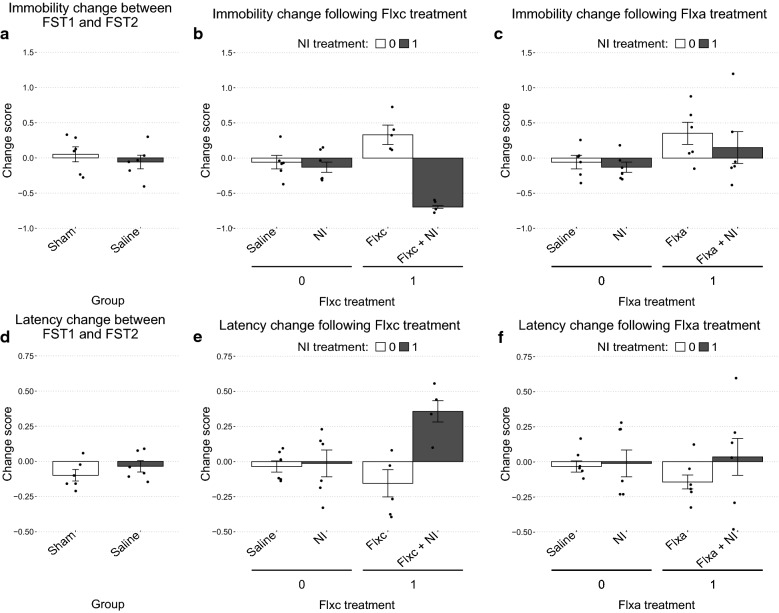
Assessing the therapeutic efficacy of the treatment combinations based on their effects on change scores of depressive symptoms between the FSTs. Change scores are calculated for each animal by applying the following formula to the relevant values: (FST2–FST1)/FST1. **a** & **d** Establishing the baseline change of depressive symptoms between FST1 and FST2 without administration of any treatments. **b** & **e** The chronic treatment setting: comparing the effects of Flxc, NI, and their combination on immobility (**b**) and latency (**e**) times. **c** & **f** The acute treatment setting: comparing the effects of Flxa, NI, and their combination on immobility (**c**) and latency (**f**) times. **a** & **d** Change scores were compared using the Welch’s t-test and no statistically significant differences were found. **b–c** & **e–f** While change scores are shown in the plots for easier interpretation, the data was actually analyzed using two-way ANCOVA using values from FST1 as the covariate. Details of this analysis are presented in the main text

Furthermore, we compared the amount of change from FST1 to FST2 between the two groups. As the amount of change was affected by the base value at FST1 (the covariate), we used the change scores as defined in the Methods section. The amount of change did not differ significantly between the Sham and Saline groups in neither immobility (Sham: mean = 0.05, SD = 0.26 / Saline: mean = − 0.05, SD = 0.24; *t*(9.9) = 0.76, *p* = 0.5, *d* = 0.44) (Fig. 2a) nor latency (Sham: mean = − 0.1, SD = 0.1 / Saline: mean = − 0.03, SE = 0.1; *t*(10) = − 1.1, *p* = 0.3, *d* = − 0.64) (Fig. 2d).

As a result, we concluded that repeating the FSTs did not significantly alter depressive symptoms, especially in the Saline group to which the treatment combinations were compared.

#### Assessment of the therapeutic efficacy of treatment combinations on depressive symptoms

The therapeutic efficacy of the drug combinations were assessed in two general groups: the chronic setting, where the interaction of Flxc and NI was studied; and the acute setting, where the interaction of Flxa and NI was studied. In the chronic treatment setting, there was a significant main effect for the covariate of FST1 immobility time (*F*(1,16) = 31.07, *p* < 0.0001, ${\eta }_{p}^{2}$ = 0.66). Furthermore, there was a significant interaction between Flxc and NI (*F*(1,16) = 27.55, *p* < 0.0001, ${\eta }_{p}^{2}$ = 0.63). Interpretation of the interaction using simple effect analysis with orthogonal contrasts determined that NI showed close to no effect on immobility times in the absence of Flxc (Saline: mean = 62.25, SD = 11.94 / NI: mean = 56.50, SD = 12.01; *d* = − 0.48, *p* = 0.418). In contrast, administration of NI alongside Flxc dramatically decreased immobility (Flxc: mean = 78.57, SD = 12.69 / Flxc + NI: mean = 15.16, SD = 12.50; *d* = − 5.03, *p* < 0.0001) (Fig. 2b).

A similar pattern was observed for latency times as there was a significant interaction between Flxc and NI (*Q* = 5.47, *p* = 0.044). The change in latency times between FST1 and FST2 did not differ substantially between the NI (mean = − 0.01, SD = 0.02) and Saline (mean = − 0.03, SD = 0.10) groups (d = 0.12). However, Flxc in combination with NI was much more effective than Flxc alone in improving latency times (Flxc + NI: mean = 0.36, SD = 0.15 / Flxc: mean = − 0.15, SD = 0.22; d = 2.66) (Fig. 2e).

Compared to the chronic treatment setting, much smaller changes in immobility and latency times were observed in the acute setting. For immobility, a significant main effect for the covariate of FST1 immobility time (*F*(1,19) = 11.64, *p* = 0.0029, ${\eta }_{p}^{2}$ = 0.38) was observed. However, the interaction between Flxa and NI was not significant (*F*(1,19) = 0.32, *p* = 0.5784, ${\eta }_{p}^{2}$ = 0.02). Nevertheless, Flxa had a significant main effect (*F*(1,19) = 6.33, *p* = 0.0209, ${\eta }_{p}^{2}$ = 0.25) by increasing immobility times. The main effect of NI, however, was not significant (*F*(1,19) = 1.55, *p* = 0.2279, ${\eta }_{p}^{2}$ = 0.07) (Fig. 2c).

As for latency, the interaction between Flxa or NI (*Q* = 0.59, *p* = 0.465) was not significant. This was despite the large effect size of the difference in latency change between the Flxa + NI and Flxa groups (Flxa + NI: mean = 0.14, SD = 0.04/ Flxa: mean = 0.33, SD = 0.31; d = − 4.39) compared to the NI and Saline groups (NI: mean = − 0.13, SD = 0.18 / Saline: mean = − 0.05, SD = 0.24; d = − 0.35). The main effects of Flxa (*Q* = 0.19, *p* = 0.675) and NI (*Q* = 1.04, *p* = 0.335) were not significant as well (Fig. 2f).

Based on these behavioral tests, only the Flxc + NI combination was able to significantly attenuate depressive symptoms while neither one of Flxc, Flxa, and NI elicited improvement by themselves. Notably, while the Flxa + NI combination also did not produce statistically significant changes, it substantially improved latency times.

#### Determination of the molecular machinery underlying the observed therapeutic effects

**Fig. 3:**
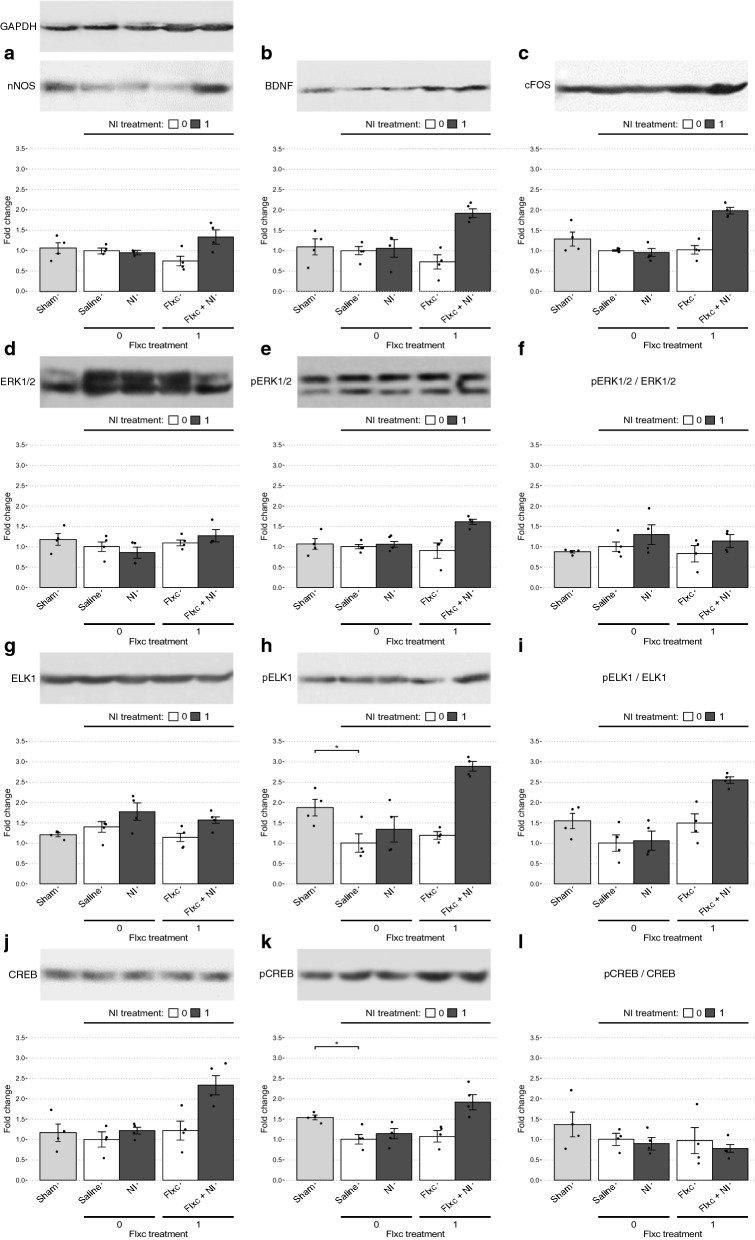
Analysis of the protein changes underlying the observed therapeutic effects in the behavioral assays. **a–l** For each marker, representative bands from the western blot studies are shown when applicable. Band intensities were corrected based on their corresponding GAPDH expression (**a**) and the fold change of all markers were calculated compared to the Saline group. The Sham and Saline groups are compared using the Welch’s t-test to determine the changes associated with EAD development and statistical significance is shown as follows: *p < .05. Afterwards, the Saline, NI, Flxc, and Flxc + NI groups are compared using two-way ANOVA to determine the changes brought about by the treatments. Details of the results of this analysis are presented in the main text

**Fig. 4:**
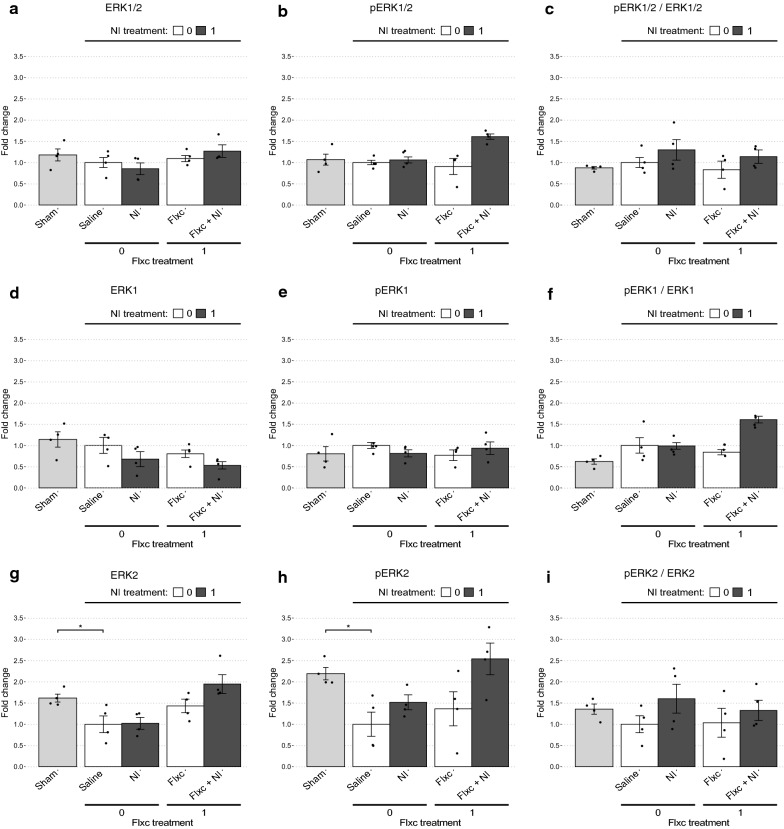
Comparison of the change profiles of ERK1/2, ERK1, and ERK2. **a–i** For each marker, band intensities were corrected based on their corresponding GAPDH expression and their fold changes were calculated compared to the Saline group. The Sham and Saline groups are compared using the Welch’s t-test to determine the changes associated with EAD development and statistical significance is shown as follows: *p < .05. Afterwards, the Saline, NI, Flxc, and Flxc + NI groups are compared using two-way ANOVA to determine the changes brought about by the treatments. Details of the results of this analysis are presented in the main text

Since significant symptomatic improvement was only observed in the chronic treatment setting, the Saline, NI, Flxc, and Flxc + NI groups were further analyzed to identify the underlying molecular mechanisms. In comparison of the Saline group to the Sham group using the t-test, only ERK2 (*p* = 0.043; Fig. 4g), pERK2 (*p* = 0.017; Fig. 4h), pCREB (*p* = 0.012; Fig. 3k), and pELK1 (*p* = 0.028; Fig. 3h) were significantly different. Of the statistically insignificant changes, pELK1/ELK1 (*p* = 0.095; Fig. 3i) and pERK1/ERK1 (*p* = 0.123; Fig. 4f) showed a relatively substantial decrease and increase, respectively.

For comparing the Saline, NI, Flxc, and Flxc + NI groups we used type III two-way ANOVA. A significant positive interaction (synergism) was observed between the Flxc and NI treatments on nNOS (*p* = 0.017; Fig. 3a), pERK1/ERK1 (*p* = 0.004; Fig. 4f), pERK1/2/ERK1/2 (*p* = 0.012; Fig. 3f), pELK1 (*p* < 0.001; Fig. 3h), pELK1/ELK1 (*p* = 0.025; Fig. 3i), CREB (*p* = 0.039; Fig. 3j), pCREB (*p* = 0.033; Fig. 3k), BDNF (*p* = 0.003; Fig. 3b), and cFOS (*p* < 0.001; Fig. 3c). For all these proteins and phosphorylation ratios (referred to collectively as markers from here onwards), NI significantly increased the fold change only in the presence of chronic fluoxetine (*p* < 0.004) and was not able to effect significant change alone (*p* > 0.11). Complementary to the simple effect analyses, post hoc tests revealed that except for nNOS, all the aforementioned markers were significantly increased in the Flxc + NI group compared to the Saline group.

In the markers which a significant statistical interaction was not observed, pERK2 (*p* = 0.022; Fig. 4h) and ELK1 (p = 0.014; Fig. 3g) had a significant main effect for NI. The main effect for NI was also close to significant for ERK1 (*p* = 0.06; Fig. 4d). Moreover, the main effect for Flxc was significant in ERK2 (*p* = 0.003; Fig. 4g) and very close to significant in pERK2 (*p* = 0.052; Fig. 4h) and ERK1/2 (*p* = 0.061; Fig. 3d). For these markers, i.e., ERK2, pERK2, and ELK1, post hoc tests revealed a significant increase in the Flxc + NI group compared to the Saline group. A more complete overview of the results of statistical analyses in this section is presented in Table 2. Furthermore, Table 3 summarizes the significant changes along with their direction of change to provide a clearer picture.

**Table 2:** The statistically significant (and close to significant) differences observed between the drug combinations in their effect on molecular markers

Marker	Saline vs Sham (t-test)	Saline vs NI vs Flxc vs Flxc + NI					
		Significant interaction?	Simple effect analysis following significant interaction:		If interaction non-significant:		Pairwise t-test with Bonferroni adjustment
			Saline vs NI	Flxc vs Flxc + NI	Significant main effect for Flxc?	Significant main effect for NI?	
nNOS	–	Yes; *F*(1,12) = 7.6, *p* = .0174, $\omega_{{}}^{2}$ = .23	Saline: mean = 1, SD = .11 / NI: mean = .97, SD = .13; *d* = .21, *p* = .78	Flxc: mean = .75, SD = .24 / Flxc + NI: mean = 1.32, SD = .34; *d* = 1.96, *p* = .004	–	–	*Flxc + NI significantly different from Flxc (*p* = .02)
ERK1	–	–	–	–	–	Close; *F*(1,12) = 4.3, *p* = .0602, $\omega_{{}}^{2}$ = .2	–
pERK1	–	–	–	–	–	–	–
pERK1/ERK1	Close, *p* = .123, *d* = 1.41	Yes; *F*(1,12) = 12.48, *p* = .0041, $\omega_{{}}^{2}$ = .42	Saline: mean = 1, SD = .36 / NI: mean = .98, SD = .17; *d* = .06, *p* = .94	Flxc: mean = .85, SD = .1 / Flxc + NI: mean = 1.6, SD = .18; *d* = 5.1, *p* = .0004	–	–	*Flxc + NI significantly different from Saline (*p* = .013), NI (*p* = .011), and Flxc (*p* = .002)
ERK2	Yes; *p* = .043, *d* = 2.01	–	–	–	Yes; *F*(1,12) = 13.83, *p* = .0029, $\omega_{{}}^{2}$ = .42	–	Flxc + NI significantly different from NI (*p* = .02) and Saline (*p* = .02)
pERK2	Yes; *p* = .017, *d* = 2.63	–	–	–	Close; *F*(1,12) = 4.65, *p* = .052, $\omega_{{}}^{2}$ = .14	Yes; *F*(1,12) = 6.93, *p* = .0219, $\omega_{{}}^{2}$ = .23	Flxc + NI significantly different from Saline (*p* = 0.03)
pERK2/ERK2	–	–	–	–	–	–	–
ERK1/2	–	–	–	–	Close; *F*(1,12) = 4.28, *p* = .0608, $\omega_{{}}^{2}$ = .17	–	–
pERK1/2/ERK1/2	–	Yes; *F*(1,12) = 8.77, *p* = .0119, $\omega_{{}}^{2}$ = .2	Saline: mean = 1, SD = .1 / NI: mean = 1.06, SD = .17; *d* = .48, *p* = .7	Flxc: mean = .9, SD = .38 / Flxc + NI: mean = 1.61, SD = .1; *d* = 2.51, *p* = .0006	–	–	*Flxc + NI significantly different from Saline (*p* = .011), NI (*p* = .023), and Flxc (*p* = .004)
ELK1	–	–	–	–	–	Yes; *F*(1,12) = 8.2, *p* = .0143, $\omega_{{}}^{2}$ = .3	Difference between Flxc and NI close to significant (*p* = .05)
pELK1	Yes; *p* = .028, *d* = 2.03	Yes; *F*(1,12) = 10.87, *p* = .0006, $\omega_{{}}^{2}$ = .15	Saline: mean = 1, SD = .45 / NI: mean = 1.34, SD = .62; *d* = .62, *p* = .27	Flxc: mean = 1.19, SD = .19 / Flxc + NI: mean = 2.88, SD = .24; *d* = 7.92, *p* < .0001	–	–	*Flxc + NI significantly different from Saline (*p* < .001), NI (*p* = .001), and Flxc (*p* < .001)
pELK1/ELK1	Close; *p* = .095, *d* = 1.40	Yes; *F*(1,12) = 6.55, *p* = .025, $\omega_{{}}^{2}$ = .11	Saline: mean = 1, SD = .4 / NI: mean = .97, SD = .48; *d* = .83, *p* = .11	Flxc: mean = 1.05, SD = .49 / Flxc + NI: mean = 2.55, SD = .13; *d* = 4.22, *p* = .0024	–	–	*Flxc + NI significantly different from Saline (*p* < .001), NI (*p* < .001), and Flxc (*p* = .01)
CREB	–	Yes; *F*(1,12) = 5.36, *p* = .0392, $\omega_{{}}^{2}$ = .09	Saline: mean = 1, SD = .37 / NI: mean = 1.22, SD = .17; *d* = .87, *p* = .45	Flxc: mean = 1.22, SD = .49 / Flxc + NI: mean = 2.35, SD = .48; *d* = 2.31, *p* = .0016	–	–	*Flxc + NI significantly different from Saline (*p* = .002), NI (*p* = .009), and Flxc (*p* = .01)
pCREB	Yes; *p* = .012, *d* = 2.91	Yes; *F*(1,12) = 5.81, *p* = .0328, $\omega_{{}}^{2}$ = .12	Saline: mean = 1, SD = .25 / NI: mean = 1.12, SD = .25; *d* = .87, *p* = .508	Flxc: mean = 1.07, SD = .26 / Flxc + NI: mean = 1.92, SD = .38; *d* = 2.31, *p* = .0015	–	–	*Flxc + NI significantly different from Saline (*p* = .005), NI (*p* = .016), and Flxc (*p* = .009)
pCREB/CREB	–	–	–	–	–	–	–
BDNF	–	Yes; *F*(1,12) = 13.15, *p* = .0035, $\omega_{{}}^{2}$ = .14	Saline: mean = 1, SD = .6 / NI: mean = 1.35, SD = .57; *d* = .55, *p* = .809	Flxc: mean = .92, SD = .46 / Flxc + NI: mean = 2.47, SD = .27; *d* = 4.06, *p* = .0002	–	–	*Flxc + NI significantly different from Saline (*p* = .008), NI (*p* = .013), and Flxc (*p* = .001)
cFOS	–	Yes; *F*(1,12) = 37.04, *p* < .0001, $\omega_{{}}^{2}$ = .3	Saline: mean = 1, SD = 0 / NI: mean = .95, SD = .17; *d* = .87, *p* = .69	Flxc: mean = 1.02, SD = .22 / Flxc + NI: mean = 1.97, SD = .15; *d* = 2.31, *p* < .0001	–	–	*Flxc + NI significantly different from Saline (*p* < .001), NI (*p* < .001), and Flxc (*p* < .001)

*While performing pairwise post hoc comparisons in the presence of a significant interaction is not strictly correct from a statistical perspective, we have used such tests to give us a better understanding of the changes from the Saline group to the Flxc + NI group

**Table 3:** Summary of the most substantial changes in molecular markers relative to the Sham and Saline groups

Marker	Saline (post-SE) versus Sham	NI vs Saline	Flxc vs Saline	Flxc + NI vs Saline
nNOS	–	–	–	–
ERK1	–	–	–	–
pERK1	–	–	–	–
pERK1/ERK1	△ (*p* = .12, *d* = 1.41)	–	–	▲
ERK2	▼	–	–	▲
pERK2	▼	–	–	▲
pERK2/ERK2	–	–	–	–
ERK1/2	–	–	–	–
pERK1/2	–	–	–	▲
pERK1/2/ERK1/2	–	–	–	–
Elk1	–	–	–	–
pElk1	▼	–	–	▲
pELK1/ELK1	▽ (*p* = .09, *d* = 1.40)	–	–	▲
CREB	–	–	–	▲
pCREB	▼	–	–	▲
pCREB/CREB	▽ (*p* = .33)	–	–	–
BDNF	–	–	–	▲
cFOS	▽ (*p* = .19)	–	–	▲

Filled arrows represent statistically significant changes (*p* < .05). All empty cells indicate comparisons with *p* > .5

#### Assessment of the oxidative state in the study groups

**Fig. 5:**
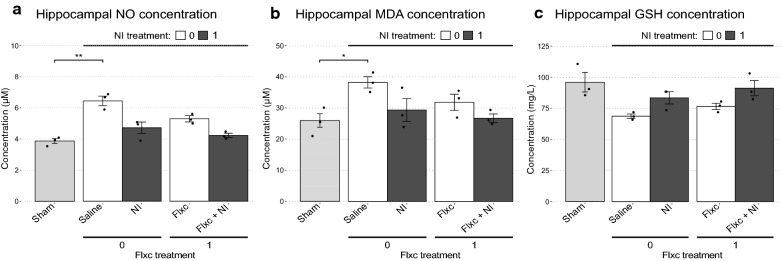
Comparison of oxidative stress markers between the study groups. **a** Comparison of nitric oxide concentrations. **b** Comparison of malondialdehyde concentrations. **c** Comparison of reduced glutathione concentrations. **a–c** The Sham and Saline groups are compared using the Welch’s t-test to determine the changes associated with EAD development and statistical significance is shown as follows: *p < .05, **p < .01. Afterwards, the Saline, NI, Flxc, and Flxc + NI groups are compared using two-way ANOVA to determine the changes brought about by the treatments. Details of the results of this analysis are presented in the main text

As a possible mediator of the synergistic effects of NI and fluoxetine, we studied the hippocampal oxidative state in the study groups using 3 markers of oxidative stress. First, the Sham and Saline groups were compared to determine how indicators of oxidative stress were changed in association with EAD development. Expectedly, NO (*t*(3.86) = 4.36, *p* = 0.013, *d* = 3.56) and MDA (*t*(2.93) = 7.48, *p* = 0.005, *d* = 6.10) levels were significantly increased in the Saline group. In contrast, GSH levels were substantially decreased in the EAD rats (*t*(2.20) = 3.41, *p* = 0.067, *d* = − 2.57). All three changes were indicative of an increase in oxidative stress in the hippocampi of post-SE rats (Fig. 5).

The effects of the treatment combinations on this increased oxidative stress were compared using two-way ANOVA. Flxc and NI did not produce a statistically significant interaction on any of the markers (NO: *F*(1,8) = 1.50, *p* = 0.256, ${\omega }^{2}$ = 0.02; MDA: *F*(1,8) = 0.55, *p* = 0.480, ${\omega }^{2}$ = 0.006; GSH: *F*(1,8) = 0, *p* = 1.00, ${\omega }^{2}$ = − 0.03). Nevertheless, both Flxc (*F*(1,8) = 9.32, *p* = 0.016, ${\omega }^{2}$ = 0.19) and NI (*F*(1,8) = 26.96, *p* = 0.001, ${\omega }^{2}$ = 0.57) showed significant main effects on NO. Furthermore, post hoc analysis showed that NO levels were decreased significantly in the NI (p = 0.011) and Flxc + NI ((*p* = 0.02) groups compared to Saline rats which was expected due to the inhibitory function of NI on nNOS.

In contrast, only NI showed a significant main effect on MDA (*F*(1,8) = 7.68, *p* = 0.024, ${\omega }^{2}$ = 0.35) and GSH (*F*(1,8) = 12.11, *p* = 0.008, ${\omega }^{2}$ = 0.47). Moreover, only the differences between the Flxc + NI and Saline groups were substantial (MDA: *p* = 0.073; GSH: *p* = 0.033). Based on these findings, NI was clearly the more potent modulator of oxidative stress and was able to reverse changes of the markers to Sham levels in many instances (Fig. 5).

## Discussion

In this study, we set out to identify the molecular machinery underlying epilepsy-associated depression by analyzing the effects of various combinations of fluoxetine and 7-nitroindazole on both behavioral and molecular aspects of EAD.

In inducing our experimental model, we modified the existing epilepsy-associated depression model to one with a simpler induction, lower mortality, and unchanged rates of epilepsy and EAD development. For reference, the study by Peng et al. had an SE-induced mortality rate of 64% compared to the 31.4% in our study [39]. Furthermore, these modifications did not alter the development of the characteristic hippocampal lesions found in other animal models of temporal lobe epilepsy (TLE), as shown in the histological results [39]. Therefore, use of this modified model may simplify future research on EAD.

Furthermore, in our study design, we used repeated FSTs for initial identification of the depressed epileptic animals and the subsequent measurement of antidepressant activity of drugs in a self-controlled manner. While several studies have shown repeated FSTs progressively increase depressive-like symptoms in healthy rats, they have found these symptoms to be responsive to antidepressant drugs [34, 41]. In our study, two repeated FSTs had little effect on the behavioral profile of the tested animals, especially those that were already depressed. As a result, repeated FSTs provide a robust means of assessing antidepressant activity of drugs while reducing the number of animals required for the study [34].

**Fig. 6:**
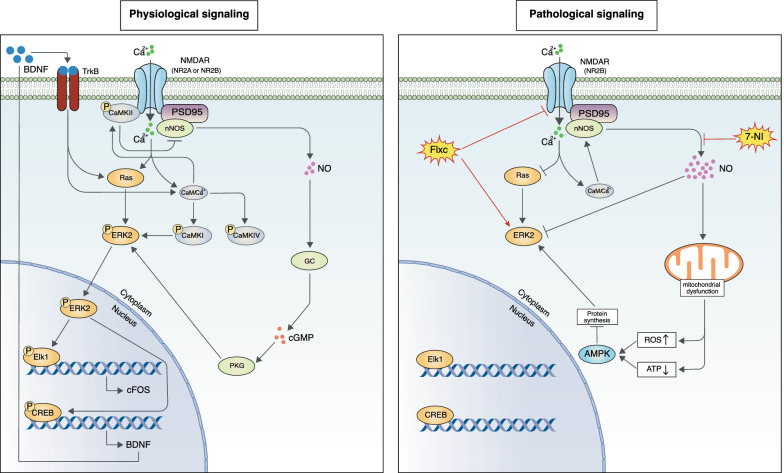
Comparison of physiological and pathological NMDAR signaling as suggested by the findings in this study. Long-term pathological changes following excitotoxic injury result in the predominance of the NR2B subunit of NMDAR which inhibits downstream ERK signaling upon overstimulation. Furthermore, these long-term changes lead to the constitutive activity of nNOS and increased NO levels, resulting in mitochondrial impairment, inhibition of protein synthesis, and direct inhibition of ERK signaling through nitrosylation

Using this study design, we determined that subeffective doses of 7-nitroindazole and chronic fluoxetine were able to positively interact to alleviate depressive symptoms in EAD. Our analysis of the possible molecular mediators of this synergism lead us to propose the pathway shown in Fig. 6 to underlie this interaction. In the following, we will discuss our logic in proposing this pathway.

When analyzing the interactions between the proteins in this pathway it is paramount to distinguish physiological and pathological signaling (Fig. 6). As mentioned before, in the physiological state, NMDAR activates Ras/ERK/CREB/BDNF signaling in NO-dependent and independent manners. However, in the pathological state, NMDAR directly inhibits this pathway [52]. Furthermore, constitutive activity of nNOS leads to chronically increased NO concentrations which results in mitochondrial impairment leading to reactive oxygen species (ROS) formation. This combination of oxidative and nitrosative stress further impairs mitochondrial function and increases apoptosis [4, 9, 51, 58]. This is corroborated by the results of the TUNEL assay in our study which indicated that apoptosis rates remained high in epileptic rats long after the initial SE injury.

Further evidence for this chronic switch from physiological to pathological signaling is provided by comparison of the expression and phosphorylation profiles of proteins between Sham and post-SE rats. In post-SE rats, ERK2 (i.e., total ERK2) and pERK2 were decreased while their ratio remained constant. This pattern is predominantly consistent with regulation at the gene expression level based on the changes of total protein. In contrast, the unchanged levels of ELK1 (i.e., total ELK1) and CREB (i.e., unphosphorylated CREB) alongside the reductions in pELK1 and pCREB signified regulation mostly by upstream phosphorylation activity and not at the level of gene expression. Despite statistical insignificance, the downward trending levels of pCREB/CREB and pELK1/ELK1 in the post-SE rats support these interpretations. Importantly, these changes were in line with our initial hypothesis, where we expected EAD to be associated with downregulation of ERK/CREB/ELK due to the various factors mentioned in the Introduction section.

Upon treatment with the Flxc + NI combination, these changes were reversed. Levels of both ERK2 and pERK2 increased while their ratio remained unchanged. In contrast, ELK1 levels remained constant despite increases in pELK1 and pELK1/ELK1. Once again, these changes were consistent with regulation predominantly at the expression and phosphorylation levels, respectively. Interestingly, CREB showed a different pattern of regulation to its initial change post SE, here showing a pattern similar to that of ERK2 rather than ELK1. Expectedly, these changes resulted in significant rises in BDNF and cFOS. In stark contrast to these proteins, ERK1 (i.e., total ERK1) was slightly decreased while pERK1 remained constant, resulting in a significant increase in the pERK1/ERK1 ratio. Interestingly, unlike ERK2, CREB, and ELK1, the increase in pERK1/ERK1 upon Flxc + NI treatment was not a reversal of the changes observed in post-SE rats compared to the Sham group as the ratio increased in both instances.

Before discussing how these changes may be brought about by Flxc + NI administration, we will first focus on some of the more interesting patterns observed in the protein expression and activation profiles. One notable finding was the difference in change patterns between ERK1, ERK2, and the aggregate ERK1/2 (Fig. 4). Even though there is some evidence that ERK1 and ERK2 each may have specific roles, it is generally accepted that they are functionally redundant and that global ERK1/2 has greater biological significance than either kinase individually. Furthermore, it has been proposed that the observations of contrasting activities of the two kinases may be attributed to their differential expression levels (and, hence, compensatory capacities) rather than their differential activity [11, 27, 32]. Nevertheless, in our study, ERK1 and ERK2 appeared to be differently regulated, with ERK2 seeming to be the major contributing factor in both the emergence of EAD and its alleviation by Flxc + NI treatment. Notably, the exact mirroring of the changes of depressive symptoms in the changes of ERK2 and the proteins downstream to ERK (i.e., ELK1 and CREB) is concealed when the kinases are inspected together as ERK1/2. While the potential for functionally relevant inference from such data is quite limited, these results are suggestive of differential regulation and function for ERK1 and ERK2.

Another notable finding is how decreases and increases in ELK1 activity were correlated inversely with depressive symptoms. This is in contrast with the previous study by Apazoglou et al. where ELK1 activity had been associated with depression [3] and is more in line with studies showing concurrent regulation of CREB and ELK1 by ERK [13, 57]. This discrepancy may be attributed to differences in the molecular alterations underlying the depressive symptoms, since depression followed excitotoxic neuronal damage in our study. Another explanation may be the biphasic pro-apoptotic and anti-apoptotic activity of ELK1 based on its subcellular localization [5].

Upon deeper inspection of the data, these changes could be linked to various known functions of fluoxetine and NI. As for fluoxetine, the following three effects may be considered. First, fluoxetine has been shown to directly inhibit NMDAR, with this effect being implicated in the analgesic effects of SSRIs [6, 53]. In our study, inhibiting the inhibitory NMDAR may have contributed to the activation of its downstream pathway. Furthermore, as previously mentioned, fluoxetine has been shown to increase ERK/CREB activity through various pathways [2, 50, 54]. Finally, fluoxetine has been shown to decrease oxidative stress [1, 56], a function which we also found to some degree in our study.

On the other hand, reduction of NO to physiological levels by NI may reinstate the NO/GC/cGMP/PKG/ERK pathway. Interestingly, this pathway has been shown to directly regulate the phosphorylation of ELK1 but not CREB, rather activating the latter through its coactivator, TORC1 [18]. This differential action aligns nicely with the regulatory differences observed between the two transcription factors in our study. Furthermore, the effects of NI may be mediated through a decrease in protein S-nitrosylation. Multiple studies have shown nitrosylation-dependent downregulation of ERK activity, either directly or through modulation of upstream proteins [15, 22, 43, 47]. In addition, nitrosylation has been shown to rapidly reverse upon reduction of NO levels [10], making it a suitable candidate for mediating the rapid antidepressant effects observed in this study.

Another pathway for NI’s effects may have been its ability to decrease the oxidative state present in post-SE hippocampi. Interestingly, the combined effects of NI and Flxc on oxidative stress was additive rather than synergistic with NI being the more prominent player. Moreover, either NI alone or the combination of Flxc and NI was able to almost completely reverse the post-SE oxidative changes to Sham levels. The relevance of the oxidative state to our proposed pathway lies in the fact that reduced ATP and increased ROS levels in cells have been shown to activate the cell metabolism regulator enzyme, AMPK. Upon activation, this enzyme inhibits proteins synthesis with the goal of shifting cell metabolism away from anabolism towards catabolism and ATP production. These inhibitory effects on protein synthesis are mediated by mTORC1 [25]. Since increased expression of multiple proteins was observed only following the Flxc + NI combination when oxidative stress markers reached almost normal levels, this might explain why neither fluoxetine nor NI were able to increase ERK2 and CREB expression by themselves.

To summarize, a general mechanism of simultaneous inhibition removal (Flxc on NMDAR, NI on oxidative stress, NI on nitrosylation) and activation (ERK/CREB/ELK1 activation by Flxc through CaMKIV and 5HT1a, physiological concentrations of NO through cGMP/PKG, and physiological NMDAR signaling) may explain the synergistic effects of NI and fluoxetine. The BDNF produced by this pathway could then maintain and amplify this activity through its TrkB receptor [62].

## Conclusions

In conclusion, we propose anti-nNOS treatment as a viable option to overcome SSRI-resistance in epilepsy-associated depression. Furthermore, we suggest the pathways shown in Fig. 6 to mediate the development of EAD and its alleviation by Flxc + NI treatment. Indeed, we realize that our proposed pathway requires further validation in other models of EAD.

Based on our findings, we propose several prospects for future studies. First, clinical characterization of selective nNOS inhibitors and NMDAR/nNOS uncouplers could unlock their potential as powerful treatments for conditions in which excitotoxicity plays an important pathophysiological role, such as status epilepticus, traumatic brain injury, and cerebral ischemia [63]. Second, while the pathway proposed in this study combined elements of both epileptogenesis and formation of depressive symptoms, other animal models of depression could be studied to determine whether this pathway also underlies depression when excitotoxicity plays a more limited role. Importantly, researchers studying this pathway must be mindful of whether they are targeting its physiological or pathological state. To mention some frequent methods targeting this pathway, use of NO donors, NMDAR agonists, calcium ionophores, and their concentrations and durations of action (acute vs. chronic) would be important determinants of the pathway’s response to stressors and treatments. Third, while we observed an increase in BDNF and cFOS production through the ERK/CREB/ELK1 pathway, caution must be exercised as this state is, as previously mentioned, also observed immediately after seizures and other types of neuronal damage, leading to aberrant neurogenesis and epileptogenesis [20, 36]. Therefore, it could be studied whether the therapeutic effects observed in our study are also associated with aberrant neurogenesis and if they are followed by exacerbations in the underlying epilepsy or its associated depression in the long term. Finally, the possibility of differential functions of ERK1 and ERK2 could be studied, possibly within the context of their gene expression and associations with adaptor proteins.

## Data Availability

All data generated or analyzed during this study are included in this published article.

## Abbreviations

BDNF: brain-derived neurotrophic factor
CaMKII: calcium/calmodulin-dependent kinase II
CREB: cAMP-response element binding protein
DG: dentate gyrus
EAD: epilepsy-associated depression
ERK: extracellular-regulated kinase
Flxa: acute fluoxetine treatment
Flxc: chronic fluoxetine treatment
FST: forced swim test
GC: guanylyl cyclase
GSH: reduced glutathione
IEG: immediate early gene
i.m.: intramuscular
i.p.: intraperitoneal
LFP: local field potential
LTP: long-term potentiation
MDA: malondialdehyde
NI: 7-nitroindazole
NMDAR: N-methyl-D-aspartate receptor
nNOS: neuronal nitric oxide synthase
NO: nitric oxide
PKG: cGMP-dependent protein kinase
ROS: reactive oxygen species
s.c.: subcutaneous
SE: status epilepticus
TBA: thiobarbituric acid
